# Metagenomics Reveals Specific Microbial Features in Males with Semen Alterations

**DOI:** 10.3390/genes14061228

**Published:** 2023-06-06

**Authors:** Iolanda Veneruso, Federica Cariati, Carlo Alviggi, Lucio Pastore, Rossella Tomaiuolo, Valeria D’Argenio

**Affiliations:** 1 Department of Molecular Medicine and Medical Biotechnologies, Federico II University, Via Sergio Pansini 5, 80131 Napoli, Italy; 2 CEINGE-Biotecnologie Avanzate Franco Salvatore, Via G. Salvatore 486, 80145 Napoli, Italy; 3 Department of Public Health, Federico II University, Via Sergio Pansini 5, 80131 Napoli, Italy; 4 Faculty of Medicine, Università Vita-Salute San Raffaele, Via Olgettina 58, 20132 Milano, Italy; 5 Department of Human Sciences and Quality of Life Promotion, San Raffaele Open University, Via di Val Cannuta 247, 00166 Roma, Italy

**Keywords:** infertility, male infertility, semen microbiota, metagenomics

## Abstract

Infertility incidence is rising worldwide, with male infertility accounting for about 50% of cases. To date, several factors have been associated with male infertility; in particular, it has been suggested that semen microbiota may play a role. Here, we report the NGS-based analyses of 20 semen samples collected from men with (Case) and without (Control) semen alterations. Genomic DNA was extracted from each collected sample, and a specific PCR was carried out to amplify the V4-V6 regions of the 16S rRNA. Sequence reactions were carried out on the MiSeq and analyzed by specific bioinformatic tools. We found a reduced richness and evenness in the Case versus the Control group. Moreover, specific genera, the *Mannheimia*, the *Escherichia_Shigella,* and the *Varibaculum,* were significantly increased in the Case compared to the Control group. Finally, we highlighted a correlation between the microbial profile and semen hyperviscosity. Even if further studies are required on larger groups of subjects to confirm these findings and explore mechanistic hypotheses, our results confirm the correlation between semen features and seminal microbiota. These data, in turn, may open the way to the possible use of semen microbiota as an attractive target for developing novel strategies for infertility management.

## 1. Introduction

Infertility incidence is progressively increasing worldwide, and it is estimated that about 50% of cases are due to male infertility [1]. Male infertility represents a highly heterogeneous condition that may be related to pre-testicular (i.e., alterations of the hypothalamic–pituitary axis), testicular, and post-testicular (i.e., urogenital obstructions, vasectomy, and accessory glands impairment) diseases [2]. Moreover, genetic, environmental, and microbiological factors have also been related to this condition [3,4,5]. The correct identification of the possible cause of male infertility, together with the female partner assessment, is crucial for proper couples’ evaluation and ensuring the best strategy to improve couples’ reproductive outcomes [6]. The semen analysis represents a routine step in assessing male infertility since it provides considerable information regarding macroscopic and microscopic features that can highlight underlying diseases [7,8]. Moreover, we have recently reported that semen sample parameters’ alterations correlate with urogenital infections and sperm DNA fragmentation, suggesting that an in-depth semen evaluation may improve male infertility management [9]. 

In recent years, metagenomics has been widely used to characterize the taxa and gene content of the human microbiome. In particular, microbiome analyses allow for estimating the taxonomic and functional composition of the different microorganisms present in selected groups of samples through transversal and longitudinal studies. In this context, it has been shown that human semen hosts a specific microbial community featured by a high inter-individual variability [4]. This semen microbiota may play a role in health reproduction by influencing spermatozoa functions. Moreover, the microbial transfer may also impact the female partner’s and their offspring’s health [4,10]. As a consequence, a semen microbiota analysis has emerged as an attractive tool to understand better the mechanisms underlying male infertility and for the development of novel therapeutic strategies that, based on microbial manipulation, may improve couples’ reproductive outcomes.

Here, we report the semen microbiota analysis of 20 subjects undergoing routine evaluations to assess couples’ infertility. Interestingly, we found different microbial features in the males with semen parameter alterations. Furthermore, we highlighted specific microbiome alterations related to semen hyperviscosity, thus highlighting a potential mechanism through which the semen microbiota may impair fertility and suggesting novel, attractive targets for therapeutic interventions.

## 2. Materials and Methods

### 2.1. Patients’ Enrollment and Sample Collection

Twenty men (age range 27–48 years), selected among those undergoing a fertility assessment at the Federico II University of Naples between February 2019 and January 2020, were included in this study. The presence of primary gonadal pathologies, primary or secondary hypogonadism, a positive history of genital surgery, radio and/or chemotherapy, and concomitant therapies were considered as exclusion criteria. The study was carried out according to Helsinki declaration rules and was approved by the local ethical committee (Federico II Ethics Committee, Number: 382-18). 

From each study subject, a semen sample was collected. The latter were obtained after 2–7 days of sexual abstinence and analyzed by standard procedures according to WHO guidelines [7]. In particular, semen samples were analyzed through macro- and microscopic evaluation, as previously reported [9]. The obtained values were compared with the references considering the lower 5th centile value as a cut-off to highlight any alteration.

### 2.2. DNA Extraction and 16S rRNA Analysis

Genomic DNA was extracted from each collected sample using a phenol chloroform-based procedure. In detail, 500 µL of Lysis Buffer (100 mM TRIS HCL pH 8.5, 5 mM EDTA, 0.2 % SDS, and 200 mM NaCl) and 25 µL of Proteinase K were added to each tube containing a pellet of a semen sample previously centrifugated at 11,000 rpm for 5 min. Samples were placed on the ThermoMixer at 55 °C and 550 rpm overnight. Then, 500 µL of phenol-chloroform was added to each tube, shaken, and centrifuged at 4 °C for 10 min at 11,000 rpm, obtaining two phases, one organic and one watery. The supernatant (watery phase) that contained the DNA was recovered. One ml of cold 100% ethanol was added to the supernatant, shaken, and centrifugated at 11,000 rpm for 10 min at 4 °C. After 2 wash steps with 800 µL of cold 75% ethanol/each, the ethanol was removed, and the pellet was dried before it was resuspended in 30 µL of molecular water. The obtained DNA samples were quantized using the nanodrop spectrophotometer (Thermo Fisher Scientific Inc., Waltham, MA, USA) before the metagenomic analysis. To minimize this risk of contaminations during this analytical step, the DNA extraction of all samples was performed in a pre-PCR designated room under a laminar-flow hood.

In particular, to simultaneously isolate the 16S rRNA gene of all the bacterial taxa present in the collected semen samples, a first-round PCR was carried out using custom primers, allowing the amplification of the V4-V6 hypervariable regions of the bacterial 16S rRNA. These custom primers included the overhang sequences with Illumina adapters; forward primer, 5′-TCGTCGGCAGCGTCAGATGTG TATAAGAGACAGCCAGCAGCCGCGGTAAT-3′; reverse primer, 5′-GTCTCGTGGGCTC GG AG ATGTGTATAAGAGACAGGGGTTGCGCTCGTTGC-3′. As previously reported, the PCR mix and amplification conditions were optimized to ensure proper amplification, avoiding forming specific products and/or primer’s dimers [11]. Two negative controls were also included in the PCR reactions and were processed with the patients’ samples to control potential environmental contaminations. After the 2% agarose gel electrophoretic analysis, PCR amplicons’ purification was made by using the AMPure XP Beads (Beckman Coulter, Brea, CA, USA), and a quality check was carried out on the Tape Station System with the D1000 ScreenTapes (both from Agilent Technologies, Santa Clara, CA, USA).

Then, a second-round PCR was performed to add specific indexes to each sample and the universal adapters for the following NGS reactions, according to the protocol that we previously described [11,12]. Furthermore, in this step, 2 negative controls were included.

Finally, each sample was quantified using the Qubit fluorometer (Life Technologies, Carlsband, CA, USA) and diluted at 4 nM to prepare the pool to be sequenced. In particular, 9 pM of libraries’ pool was loaded with 30% of 9 pM Phix. Sequencing reactions were carried out on the Illumina MiSeq System, using 2 MiSeq Reagent Nano Kit V2 500 cycles (Illumina, San Diego, CA, USA).

### 2.3. Bioinformatic Analysis

The CEINGE Biotecnologie Avanzate Franco Salvatore Bioinformatic Facility analyzed the FASTQ files generated by the sequencing runs. In particular, sequences have been checked for quality by using FastQC and aligned against the reference database SILVA NR 99 v.138 to assign OTUs (operational taxonomic units) correctly. The OTU table and the taxonomy table have been used for further analyses through the web-based tool Microbiome Analyst (2.0, last accession in March 2023) [13]. In particular, α diversity was measured using different metrics to assess both richness and evenness; the ANOVA test was applied to evaluate statistically significant differences. Unweighted and weighted UniFrac distance measures were coupled with the PERMANOVA test to evaluate any significant differences in the β diversity. A differential abundance analysis was evaluated using a univariate statistical test based on the DESeq2 algorithm; *p*-values were adjusted using the FDR method. The Tax4Fun pipeline was used for functional capabilities’ prediction using SILVAngs as an annotation tool. The MaAsLin2 package was used for the multivariable association between clinical data and microbiome features (adjusted *p*-value cutoff: 0.05) [14].

## 3. Results

A standard semen samples analysis allowed us to classify the study subjects as “Case” or “Control” groups based on the presence/absence of alterations. Consequently, 13/20 subjects fell within the Case group and 7/20 within the Control group (Table 1). 

All these samples were sequenced to investigate their bacterial composition, as described deeply in the Methods. An average of 47,660 reads/sample were obtained, corresponding to 859 identified OTUs. The negative controls in the experimental procedure received no reads; thus, potential environmental contaminations were excluded and not included in the downstream analyses. 

**Figure 1:**
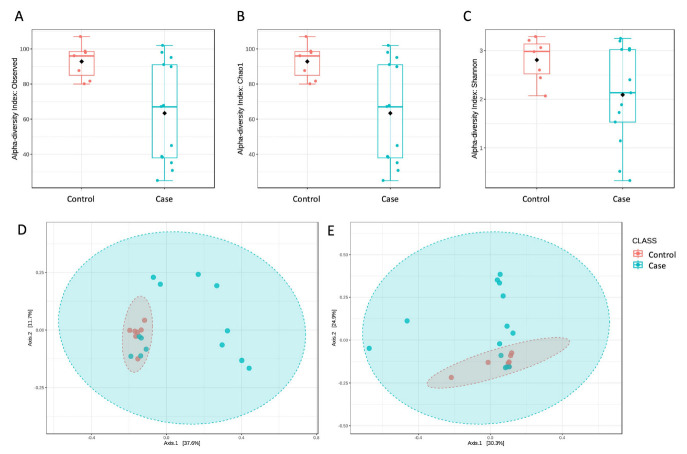
Diversity measures analyses highlighted significant differences between the two tested conditions. In particular, we found that men with semen parameters’ alterations (Case group) had reduced biodiversity of their bacterial metagenome, as assessed by both Observed species ((**A**), *p* = 0.004) and Chao1 ((**B**), *p* = 0.004) metrics. Moreover, this group was also featured by a significantly reduced evenness, as measured by the Shannon index ((**C**), *p* = 0.04), indicating a low proportion between taxa. Β diversity was also evaluated by using the unweighted (**D**) and weighted (**E**) UniFrac distance measures. Statistical significance was assessed by the PERMANOVA test, resulting in significance (*p* = 0.02) only in the case of unweighted UniFrac.

Diversity analyses showed significant differences between the two study groups. First, we evaluated α diversity to assess richness (i.e., the number of taxa present in a group) and evenness (i.e., the representation of each taxon within a group). Interestingly, neither richness, evaluated by the Observed species (Figure 1A) and Chao1 (Figure 1B) measures, or evenness, assessed by the Shannon index (Figure 1C), were significantly different (*p* < 0.05) between the Control and Case groups. The latter group, in particular, was found to have a reduced biodiversity and a non-homogeneous representation of the taxa contributing to this community.

The β diversity analysis also highlighted a significant difference between the two groups, as assessed by an unweighted UniFrac distance measure *(p* < 0.05, Figure 1D). The weighted UniFrac distance metric (Figure 1E) was not significantly different (*p* = 0.07). This finding suggests that the variations between the two tested conditions may be due to the kind of taxa present in the microbial communities rather than their different abundances, as reported for other diseases [11,12]. It has to be noticed that the Case group showed a large heterogeneity compared to the Control one, resulting from both α and β diversity evaluations. This behavior was not related to a specific semen parameter but may be a consequence of different conditions affecting the reproductive outcome of these subjects. Nevertheless, the diversity measures showed significant differences between the two tested conditions.

**Figure 2:**
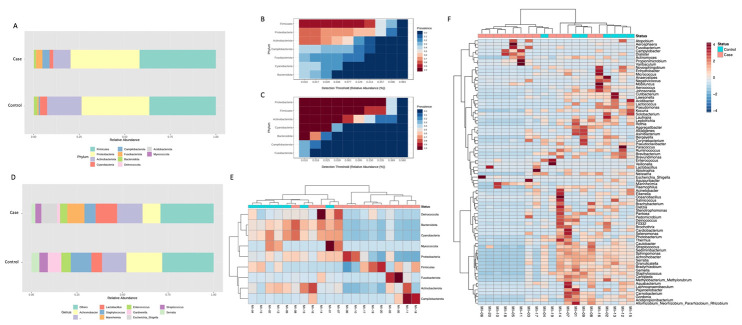
Different microbial taxa were found in Control and Case groups after taxonomic assignment. A different bacterial composition (relative abundance, %) was highlighted at the phylum level (**A**), as also confirmed by core microbiome analysis showing different sets of taxa in Case (**B**) and Control (**C**) groups. These differences in taxa composition were also present at the genus level, as highlighted in panel **(D**), reporting the top 10 represented taxa (relative abundance, %) in the two analyzed conditions. Finally, to evaluate abundance patterns, a heatmap of variance was obtained by grouping the reads according to the observed taxa. A clear cluster was obtained between the two tested groups at the phylum (**E**) and the genus levels (**F**).

The taxonomic assignment was then carried out. Ten phyla were identified, with seven showing an abundance higher than 1% in at least one of the two groups (Figure 2A). Proteobacteria were the most abundant phylum in both groups (about 37% of relative abundance in both conditions). In comparison, Firmicutes and Actinobacteria were respectively more (from 36.5% to 41.9%) and less (from 18.9% to 9.5%) abundant in the Case group compared to the Control. Moreover, Campilobacterota and Fusobacteriota were more represented in the Case group (Figure 2A).

The core microbiome analysis confirmed that a different set of taxa was identified at the phylum level in the Case (Figure 2B) and Control (Figure 2C) groups considering a relative abundance of 1% and a sample prevalence of 20%. At the genus level, 10 taxa were most represented (Figure 2D). In particular, *Achromobacter* (from 19.7% to 9.8%), *Staphylococcus* (from 11.2% to 6.1%), *Gardnerella* (from 7.3% to 1.5%), and *Serratia* (from 4.4% to 2.1%) were most abundant in the Control compared to the Case group. Instead, the *Lactobacillus* (from 5.9% to 11.8%), *Escherichia_Shigella* (<1% to 8.7%), and *Serratia* (from 4.4% to 2.1%) genera had an increased abundance in the Case group (Figure 2D). Thus, a clustering analysis was performed, showing that samples belonging to the same group had a similar abundance pattern with respect to the others (Figure 2E,F).

To highlight any significant difference between the two study groups, a differential abundance analysis was also carried out. No significant results were found at the phylum and class levels. However, six orders, six families, and three genera significantly differed between the Control and Case groups and are reported in Table 2. 

**Figure 3:**
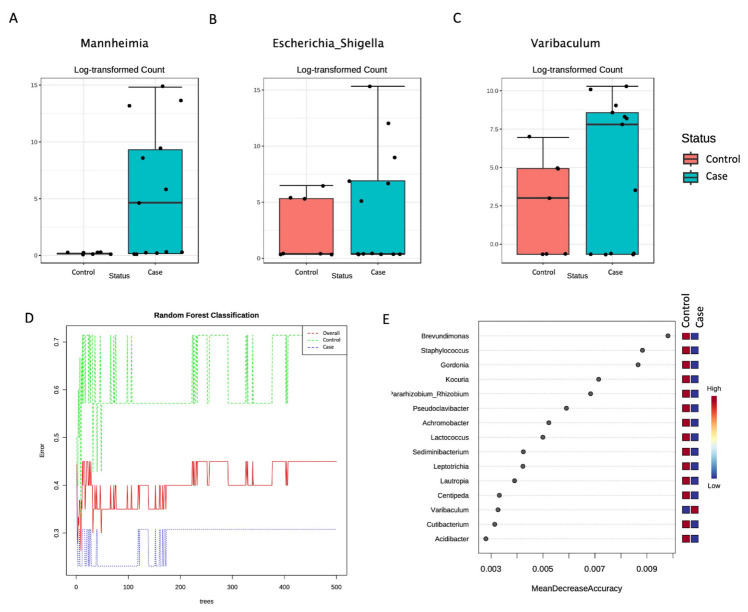
Significantly different genera were identified between Control and Case groups as measured by using differential abundance analysis coupled with the DESeq2 method (adjusted *p*-value <0.05). All three significant genera, the *Mannheimia* (**A**), the *Escherichia_Shigella* (**B**), and the *Varibaculum* (**C**), were more abundant in the Case than in the Control group. Random Forest showed different decision trees for the Case and Control groups at genus level (**D**). The features contributing to these differences are ranked based on their contribution to classification accuracy (**E**).

In particular, within the three differentially abundant genera, the *Mannheimia* (belonging to the *Pasturellales* order and the *Pasturellaceae* family), the *Escherichia_Shigella* (belonging to the *Enterobacteriaceae* family), and the *Varibaculum* (belonging to the *Actinomycetales* order) genera were all significantly increased in the Case compared to the Control group (Figure 3).

A Random Forest analysis was then applied to identify the predictive features. The generated decision trees differed for the two groups at the genus level (Figure 3D), and a list of predictive features was also generated (Figure 3E). Interestingly, all the identified genera were reduced in the Case group compared to the Control, except for the *Varibaculum* genus.

Finally, to assess the presence of a significant association between semen parameters and specific microbiome features, a multivariate analysis was performed. By analyzing all the variables together, no significant association was found at any taxonomic level. So, each semen parameter was individually analyzed. Interestingly, we found a significant association only for semen viscosity, highlighting three phyla, four classes, eleven orders, twenty families, and twenty-two genera as significantly differentially abundant in the Case compared to the Control group (Table 3). All these significant taxa were found to be less abundant in the presence of semen hyperviscosity.

## 4. Discussion

Human microbiota has been claimed as an important hint for human physiology and has rapidly emerged as a factor contributing to disease development. Indeed, a microbial counterpart has been described in almost all humans’ body sites, microbial alterations have been identified in the presence of an increasing number of diseases, and the possibility of modifying the microbiota composition by specific interventions contributes to the interest in this field [15]. 

Concerning reproduction, the increasing incidence of fertility issues is prompting research to improve the outcome of reproductive strategies [3]. In this context, both female and male reproductive systems’ microbiotas have been identified as important factors for reproductive systems’ physiology. Their alterations have been associated with pathological conditions, including infertility [4,9]. Semen microbiota alterations, in particular, have been reported as a possible cause of male infertility [4]. Thus, the identification of specific semen microbial features associated with poor reproductive outcomes may not only clarify an additional mechanism contributing to male infertility but may also open the way to novel therapeutic strategies based on semen microbiota manipulation. Here, we report the bacterial semen microbiota composition of males with (Case group) and without (Control group) alterations of semen parameters to highlight specific signatures associated with semen quality and, thus, that are able to impair fertility.

Interestingly, we found a significantly reduced richness and evenness in the Case compared to the Control group. This suggests that poor semen quality is associated with reduced bacterial biodiversity and an unequal representation of the different taxa. Reduced biodiversity is considered a general hallmark of dysbiosis [15] and has also been reported as predictive of a poor reproductive outcome: Chen et al. found that azoospermic patients had a reduced semen microbiota biodiversity and hypothesized that this, in association with the increased abundance of specific pathogenic taxa, may increase the risk of metabolic, immune, and infectious diseases [16]. Despite this significant result, β diversity analyses suggested that the differences between our study groups are explained more by a different qualitative taxa composition rather than quantitative taxa modifications. Indeed, a clustering analysis showed a good clustering between the Case and Control groups, suggesting that individuals in the same group share more microbial features than those in the other both at the phylum and genus levels. Accordingly, different taxa were identified in the two study groups at each taxonomic level, contributing to their different core microbiomes. A univariate analysis showed that three differentially abundant genera, the *Mannheimia*, the *Escherichia_Shigella,* and the *Varibaculum,* were significantly different between the two study groups, all being more abundant in the Case compared to the Control group.

*Escherichia_Shigella* has been previously reported as associated with male infertility [17]. Indeed, *Escherichia coli* was identified by culture methods in the semen samples of infertile men and was associated with a sperm motility rate reduction and increased percentage of morphological alterations [18]. Moreover, *E. coli* has been associated with reduced semen density and diminished progressive motility [19], and in vitro studies have suggested that it may impair sperm viability and motility [20,21]. However, different studies reported inconsistent results, with some noting an increased abundance in infertile men [17,22] and others not [23,24]. Interestingly, Weng et al. reported a significant increase of *E. coli* in infertile men, even if this strain was not associated with semen quality [17], according to our findings. 

*Mannheimia* strains are well-known pathogenic factors for ruminants’ respiratory diseases. So far, different virulence mechanisms have been described [25]. Moreover, *Mannheimia haemolytica* infections induce systemic inflammation, as assessed by increased serum proinflammatory cytokines. A recent study has reported alterations in semen parameters in experimental bucks challenged with *M. haemolytica* [26]. Despite the fact that this pathogen usually affects ruminants, it has been already identified in humans associated with different conditions [27,28], and it has been reported that infections in humans may occur, especially upon contact with colonized animals [29]. To the best of our knowledge, this genus has not been reported before in semen samples from humans with fertility issues. However, due to its pathogenic features and previous reports, it is suitable to suppose a pathogenic role. Further studies are required to address these issues. In particular, functional studies demonstrating its possible proinflammatory activities and effects on sperm features may allow us to define a potential role in male infertility.

Finally, the *Varibaculum* genus was enriched in prostatis [30] bladder and prostate cancers [31,32]. Interestingly, it has been already reported in the semen samples of men belonging to infertile couples [33], and Weng et al. associated its presence with altered sperm parameters in men from infertile couples [17].

Finally, the multivariate analysis highlighted a significant association between semen microbial alterations and semen viscosity. Semen hyperviscosity is a well-known factor able to impair male fertility [34]. Indeed, it can reduce sperm motility and decrease sperm count [34]. By analyzing a cohort of 89 infertility-related cases and 29 controls, Monteiro et al. found that seminal hyperviscosity and oligoasthenoteratozoospermia correlated to an increased abundance of *Neisseria*, *Klebsiella*, and *Pseudomonas* and a reduction in *Lactobacillus* [35]. Characterizing the seminal microbiota of 42 infertile idiopathic patients, Garcia-Segura et al. identified different genera in relation to seminal quality alterations, including viscosity [36]. Finally, other studies including larger cohorts of subjects were able to highlight intriguing associations between semen microbiota composition and specific semen parameters, although not with semen viscosity [37,38,39]. In our population study, 6/20 subjects had semen hyperviscosity, i.e., about half of the Case group. Interestingly, we found several taxa (from phylum to genus) to be significantly different in subjects with semen hyperviscosity, suggesting that this feature is associated with a specific microbial profile differentiating them from individuals with other semen alterations. If confirmed by further studies enrolling a larger group of patients, this finding may clarify the mechanisms involved in the relationship between male infertility and semen hyperviscosity and open the way to developing novel therapeutic strategies that, by modifying the microbiota, may positively impact semen viscosity.

Despite these promising findings, one limit of this study is the small number of analyzed subjects that may hamper the possibility to infer general conclusions. On the other hand, the partial overlap between the data presented herein and previous reports is encouraging. Further studies on large and well-characterized groups of subjects will be required to more deeply investigate the relationship between semen microbiota and male infertility.

## 5. Conclusions

In this study, the examination of the semen microbiota composition of males with and without semen alterations was carried out by an NGS-based analysis. Significant differences were highlighted between the two study groups, according to previous works suggesting a potential role of semen microbiota in male infertility. Further studies are required to confirm these findings on an increased number of subjects and to verify functional contributions. Once this is assessed, it is feasible to suppose that the microbiome analysis and consequent microbiota manipulation may become essential for male infertility management.

## Figures and Tables

**Table 1:** Semen samples’ parameters, as evaluated by standard analysis, are reported for each study group. Percentages, average values, and upper and lower values (in parenthesis) are reported for each parameter.

Semen Parameter	Case Group (N = 13)	Control Group (N = 7)	Ref. Limit *
Viscosity	>2 cm filament 46% (6/13)	<2 cm filament 100% (7/7)	<2 cm filament
pH	7.7 (7–8.2)	7.6 (7.5–7.9)	≥7.2
Volume	2.3 (1–4.4)	3 (1.3–6.3)	≥1.5 mL
Sperm concentration (×10^6^ mL)	20.2 (1.5–50)	84.6 (35–170)	≥15 × 10^6^ mL
Total sperm number (×10^6^ mL)	40 (3.75–108)	255 (45.5–418)	≥39 × 10^6^ ejaculate
Total sperm motility (PR + NP, %)	29.5 (0–55)	59.3 (40–80)	≥40%
Progressive motility (PR, %)	29.6 (0–65)	63.6 (40–85)	≥32%
Leucocytes (1 × 10^6^/mL)	2.7 (rare-10)	rare	<1 × 10^6^ mL
Sperm morphology (%)	3.4 (0–10%)	6 (4–10)	≥4% (normal forms)
Germinal cells	rare	rare	<10%
Agglutination	rare	absent	rare/absent

* Reference values are based on the lower 5th centile (95% confidence interval) according to WHO’s guidelines [7]. PR: rapid progressive; NP: non-progressive.

**Table 2:** Full list of significant taxa identified by differential abundance analysis as assessed by DESeq2 (adjusted *p*-value < 0.05) between Case and Control groups. Bacterial taxa are reported according to taxonomic rank and ordered based on *p*-values (from the most significant value).

Rank	Taxon	*p*-Value	FDR
Order	*Veillonellales_Selenomonadales*	2.8019 × 10^−5^	0.00098066
Order	*Peptostreptococcales_Tissierellales*	0.00013992	0.0024486
Order	*Pasteurellales*	0.0018522	0.021609
Order	*Actinomycetales*	0.005292	0.046305
Order	*Fusobacteriales*	0.0074557	0.047691
Order	*Campylobacterales*	0.0081756	0.047691
Family	*Peptoniphilus*	1.5837 × 10^−5^	0.00098188
Family	*Veillonellaceae*	0.00027895	0.0064464
Family	*Enterobacteriaceae*	0.00031192	0.0064464
Family	*Campylobacteraceae*	0.0015619	0.024209
Family	*Fusobacteriaceae*	0.0036006	0.039076
Family	*Pasteurellaceae*	0.0037816	0.039076
Genus	*Mannheimia*	6.6625 × 10^−29^	5.863 × 10^−27^
Genus	*Escherichia_Shigella*	0.00078541	0.034558
Genus	*Varibaculum*	0.0014709	0.043147

**Table 3:** Full list of significant taxa identified by covariate analysis as assessed by the MaAsLin2 pipeline (adjusted *p*-value < 0.05) between Case and Control groups considering semen viscosity as a covariate factor. Bacterial taxa are reported according to taxonomic rank and ordered based on *p*-values (from the most significant value).

Rank	Taxon	*p*-Value	FDR
Phylum	Actinobacteriota	0.00139	0.00837
Phylum	Bacteroidota	0.00123	0.00837
Phylum	Cyanobacteria	0.000922	0.00837
Class	*Actinobacteria*	0.00145	0.00945
Class	*Alphaproteobacteria*	0.00111	0.00945
Class	*Bacteroidia*	0.00123	0.00945
Class	*Cyanobacteriia*	0.000922	0.00945
Order	*Burkholderiales*	0.000639	0.0144
Order	*Chitinophagales*	0.00133	0.0144
Order	*Chloroplast*	0.000922	0.0144
Order	*Corynebacteriales*	0.0015	0.0144
Order	*Micrococcales*	0.00101	0.0144
Order	*Propionibacteriales*	0.00034	0.0144
Order	*Rhizobiales*	0.00169	0.0144
Order	*Sphingomonadales*	0.00116	0.0144
Order	*Caulobacterales*	0.00217	0.0148
Order	*Staphylococcales*	0.00537	0.0304
Order	*Lachnospirales*	0.00747	0.0366
Family	*Alcaligenaceae*	0.00063	0.0118
Family	*Carnobacteriaceae*	0.000349	0.0118
Family	*Comamonadaceae*	0.0012	0.0118
Family	*Gemellaceae*	0.000764	0.0118
Family	*Micrococcaceae*	0.000663	0.0118
Family	*Neisseriaceae*	0.000173	0.0118
Family	*Propionibacteriaceae*	0.00034	0.0118
Family	*Sphingomonadaceae*	0.00116	0.0118
Family	*Xanthobacteraceae*	0.00107	0.0118
Family	*Yersiniaceae*	0.00041	0.0118
Family	*Chitinophagaceae*	0.00133	0.0121
Family	*Nocardiaceae*	0.00184	0.0144
Family	*Caulobacteraceae*	0.00217	0.016
Family	*Beijerinckiaceae*	0.00247	0.0168
Family	*Corynebacteriaceae*	0.00256	0.0168
Family	*Leptotrichiaceae*	0.00276	0.0171
Family	*Burkholderiaceae*	0.00415	0.0245
Family	*Staphylococcaceae*	0.00611	0.0343
Family	*Lachnospiraceae*	0.00747	0.0372
Family	*Streptococcaceae*	0.00758	0.0372
Genus	*Neisseria*	5.28 × 10^−5^	0.00887
Genus	*Acidipropionibacterium*	0.000207	0.0115
Genus	*Cutibacterium*	0.000302	0.0115
Genus	*Granulicatella*	0.000412	0.0115
Genus	*Serratia*	0.00041	0.0115
Genus	*Kocuria*	0.000519	0.0124
Genus	*Achromobacter*	0.00063	0.0128
Genus	*Gemella*	0.000764	0.0128
Genus	*Bradyrhizobium*	0.00107	0.0164
Genus	*Sphingomonas*	0.00123	0.0173
Genus	*Sediminibacterium*	0.00146	0.0188
Genus	*Gordonia*	0.00184	0.022
Genus	*Leptotrichia*	0.00276	0.0273
Genus	*Methylobacterium_Methylorubrum*	0.00247	0.0273
Genus	*Lautropia*	0.00415	0.0387
Genus	*Corynebacterium*	0.00472	0.0405
Genus	*Lactococcus*	0.00482	0.0405
Genus	*Actinomyces*	0.00649	0.0452
Genus	*Caulobacter*	0.00659	0.0452
Genus	*Lawsonella*	0.00673	0.0452
Genus	*Staphylococcus*	0.00599	0.0452
Genus	*Streptococcus*	0.00771	0.0498

## Data Availability

Data are contained within the article.
